# The Double-Edged Sword of Immunotherapy—Durvalumab-Induced Polyendocrinopathy—Case Report

**DOI:** 10.3390/jcm13216322

**Published:** 2024-10-23

**Authors:** Olga Błażowska, Katarzyna Stróżna, Hanna Dancewicz, Przemysław Zygmunciak, Wojciech Zgliczyński, Beata Mrozikiewicz-Rakowska

**Affiliations:** 1 Faculty of Medicine, Medical University of Warsaw, 02-091 Warsaw, Polandkatarzyna.strozna1@gmail.com (K.S.);; 2 Department of Endocrinology, Centre of Postgraduate Medical Education, Marymoncka St. 99/103, 01-813 Warsaw, Poland

**Keywords:** durvalumab, ICI, polyendocrinopathy, T1DM, type 1 diabetes mellitus, thyroid insufficiency, endocrinopathy, endocrine adverse events

## Abstract

**Introduction:** Immunotherapy is one of the greatest advancements in oncological patient care. The broader the treatment application, the more common the adverse events associated with the therapy. Immune checkpoint inhibitors (ICI) are currently used in numerous malignancies. These drugs influence the immune cells’ interactions, which translates to interruption of immune evasion and increased anti-tumor activity. However, the disruption of immunological signaling pathways often leads to adverse events, such as endocrinological insufficiencies, among which thyroid is the most common. Moreover, the co-appearance of several insufficiencies has been previously described. **Case report:** A 73-year-old female treated with durvalumab due to non-small cell lung carcinoma was admitted to the emergency unit due to symptoms of ketoacidosis. She had a history of well-controlled type 2 diabetes mellitus and autoimmune thyroiditis. Laboratory results showed increased anti-GAD antibodies, while the low C-peptide level indicated type 1 diabetes mellitus. Moreover, over the course of longer observation, the patient presented with abrupt aggravation of her autoimmune thyroiditis. **Conclusions:** The new onset of endocrinological insufficiencies is a rare adverse event of immunotherapy. Clinicians must pay particular attention to any signs indicating these life-threatening conditions. In case of the appearance of any endocrinological adverse event, the close cooperation of oncologists and endocrinologists is required to enhance patients’ quality of life.

## 1. Introduction

Immune checkpoint inhibitors (ICI) have changed the therapy landscape for various types of cancers. Durvalumab is a humanized recombinant monoclonal antibody, which blocks the programmed cell death 1 receptor (PD-1) and its ligand (PD-L1) interactions [1,2]. Studies show its properties prolonging progression-free survival in non-small-cell lung cancer (NSCLC) after chemotherapy [3]. ICI-induced autoimmune diabetes (ICI-DM) is a rare endocrine adverse event, which can occur in patients receiving ICI [4]. Importantly, the life-threatening condition, diabetic ketoacidosis (DKA), is one of the clinical manifestations of ICI-induced DM. Due to the aforementioned clinical manifestations, patients require periodic medical examinations and check-ups.

## 2. Case Presentation

**Figure 1:**
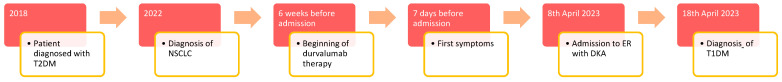
Timeline of patient’s medical history with distinguished main events. DKA—diabetic ketoacidosis; ER—emergency room; NSCLC—non-small-cell lung cancer; T1DM—type 1 diabetes mellitus; T2DM—type 2 diabetes mellitus.

A 73-year-old female with a previous history of well-controlled T2DM, diagnosed 6 years earlier and treated with metformin, and autoimmune thyroiditis visited the emergency room due to polydipsia, polyuria, and general weakness. The symptoms occurred abruptly and were observed by the patient within the last seven days. The patient started immunotherapy with durvalumab 6 weeks before her admission as a consolidation treatment for non-small cell lung carcinoma. The patient was diagnosed with NSCLC in 2022 and treated with chemotherapy (1 cycle of vinorelbine/carboplatin) at the beginning. Laboratory findings showed hyperglycemia—510 mg/dl, pH—7.14, HbA1c—8.7%, and keto- and glucosuria. Thyroid hormone levels were within normal limits. Treatment with fluids and continuous IV insulin infusion was initialized, followed by functional intensive insulin therapy. Finally, the patient’s general condition and laboratory findings improved. A glucose monitoring system was installed to monitor the patient’s glycemia continuously. Anti-GAD antibodies were detected at 1645.2 IU/mL, while the patient’s serum C-peptide level was low at 0.15 ng/mL. These laboratory findings concluded the diagnosis of T1DM, most likely related to immunotherapy. After DKA, durvalumab therapy was continued with a good response to the treatment. During further observation, the patient required adjustment of levothyroxine treatment, due to thyroid dysfunction with rapidly developing hypothyroidism (TSH 75 ug/mL). The timeline of the patient’s medical history is presented in Figure 1.

## 3. Discussion

ICIs are a great advancement in oncological care. This group of medications consists of blockers of several proteins and, to date,, the inhibitors of programmed cell death protein 1 (PD-1), programmed death-ligand 1 (PD-L1), cytotoxic T-lymphocyte-associated protein 4 (CTLA-4), and lymphocyte-activation gene 3 (LAG-3), were introduced into standards of care of numerous neoplasms, such as melanoma, non-small cell lung cancer, renal cell carcinoma, etc. [5]. The activation of T-lymphocytes requires more than one stimulation signal [6]. However, the stimulation might be interrupted by increased CTLA-4 signaling, which induces T-cell anergy [6]. Accordingly, simultaneous PD-1 and T-cell receptor (TCR) binding results in decreased phosphorylation of TCR signaling intermediates, therefore decreasing cellular activation and cytotoxic function [6]. Other immune checkpoint proteins work in a similar manner, reducing the activation of the immune response. Intriguingly, they are said to be better molecular targets for new drug development [5]. 

**Figure 2:**
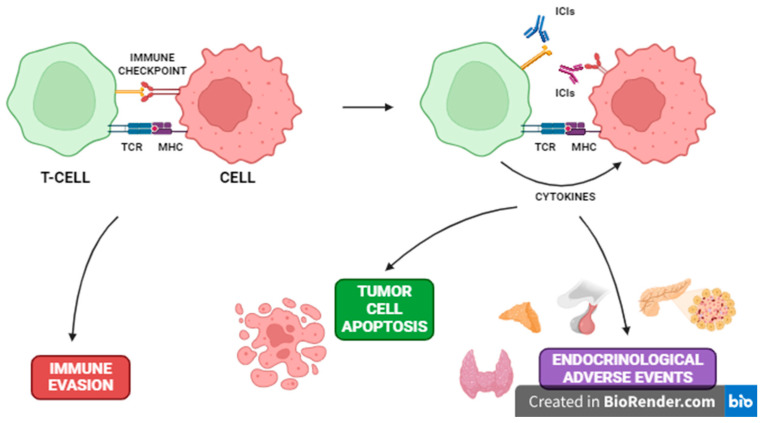
The main mechanism of ICI-induced anti-tumor and autoreactive activity. ICI—immune checkpoint inhibitor; MHC—major histocompatibility complex; TCR—T-cell receptor.

The dysregulation of T-cell response via ICIs has not only a positive anti-tumor effect, but might also lead to several immune-related adverse events (irAE) [7]. The immune-related endocrine insufficiencies were described for each ICI used in clinical practice. Several theories are trying to clarify the mechanism of the concomitant occurrence of irAEs and ICI therapy. Among them, the dysregulation of peripheral T-cell tolerance and the decreased activity of T-regulatory lymphocytes (Treg) are the most studied ones; however, the activation of autoreactive B-cells, the disruption of cytokine signaling, and microbiota imbalance may also play a role in this process [7]. Indeed, the blockage of checkpoint inhibitors, which prevent immune evasion of tumor cells, leads to the activation of autoreactive T-cell expansion, which translates into irAEs (Figure 2). However, the activation of autoantibodies after the use of ICIs is as high as 19.2%, which shows the involvement of this mechanism in irAE occurrence [8].

As mentioned before, endocrinopathies after ICI implementation develop. However, their incidence depends on the molecular target of the drug as well as the antibody itself. The incidence of the most common endocrine disruption for specific agents can be found in Table 1.

To our knowledge, this is the third case describing the onset of ICI-DM followed by hypothyroidism after durvalumab treatment. The first one described these symptoms after Bacillus Calmette–Guérin (BCG) and durvalumab therapy due to bladder cancer, whereas the second case reported a sole therapy with durvalumab for lung cancer [17,18]. Of note, this case captures the possibility of aggravating already diagnosed conditions such as T2DM and autoimmune pancreatitis due to durvalumab treatment. Importantly, durvalumab is a drug more likely to cause thyroid dysfunction than other endocrine adverse events. Almost one in ten patients treated with durvalumab experienced hypothyroidism, whereas other endocrine insufficiencies occurred in less than 1% or were not observed in the studied groups of patients [11,13].

The immune checkpoint inhibitors influence the development of diabetes mellitus, which was studied in both mice and human groups. The blockade of PD-1 or PD-L1 may cause quick onset of DM. Furthermore, PD-L1 is upregulated in beta islets exposed to inflammation [19]. In vivo studies regarding T1DM patients have shown a decrease in PD-1 gene expression in peripheral CD4+ T lymphocytes and a low level of their circulating population. On the other hand, PD-1 is expressed on regulatory T lymphocytes and plays a crucial role in their activation and therefore suppression in the immune tolerance system [20]. In a mechanism analogous to that observed in mice, inflammation increases the expression of PD-L1 on pancreatic islets in the human body. This process is triggered by IFN-gamma and CD8+ T lymphocyte infiltration [19]. Of note, the sole administration of CTLA-4 inhibitors is not able to induce ICI-DM, which is thought to be due to the lack of CTLA-4 expression in pancreatic islets [21].

Several researchers have tried to determine the classification of ICI-DM to ensure quicker diagnosis and better therapy to treat the disease. Marchand et al. proposed a classification based on the clinical presentation, distinguishing four different categories: acute diabetes with an autoimmune background, the occurrence of diabetes due to ICI-induced pancreatitis, type 2 diabetes mellitus (T2DM) phenotype-like presentation or worsening of the already-diagnosed T2DM, and diabetes following immune lipoatrophy [22]. Other researchers tried to classify ICI-DM based on the presence of diabetes-specific autoantibodies and the level of endogenous insulin production [4]. Both classifications postulate that the acute form of autoimmune diabetes presenting as fulminant diabetes is the most common one. Intriguingly, the pancreatitis group may be often associated with positive diabetes-specific antibodies as well as preserved insulin release, since, in this group, elevated lipase was commonly reported [4]. It appears that the first classification is more clinically useful, since it shows the different pathways leading to glucose metabolism disruption in ICI-treated patients. However, the second study indicates that even autoantibody-negative patients may develop diabetes, and thus should be carefully examined by physicians upon visit. 

Patients’ medical condition should be under surveillance via monitoring glucose levels, while diagnosis of diabetes mellitus, induced by ICI, is established based on C-peptide level. Treatment of ICI-induced DM depends on the patient’s C-peptide level and the simultaneous appearance of DKA. Patients with C-peptide levels appropriate for serum glucose might need lifestyle interventions, and ICI therapy can be continued. If a low level of C-peptide occurs, patients require insulin treatment, endocrine consultation, and close monitoring, while ICI-therapy should be continued [23]. The occurrence of DKA demands another way of proceeding with patients: ICI therapy should be discontinued. Furthermore, it is recommended to infuse insulin continuously, achieve and maintain correct pH and electrolyte levels, and hydrate patients intensively [23,24]. The guidelines indicate particular procedures to be followed in the case of DKA occurrence. It is customary to recommend high-dose glucocorticoid therapy for mild to severe endocrine adverse events such as hyperthyroidism or hypophysitis [25]. Interestingly, administering glucocorticoids is not advisable to treat DKA and can deteriorate glucose level control [26]. This might not be suggested due to a decrease in insulin secretion caused by GCs, which was confirmed in multiple in vitro studies. Although the exact process remains undiscovered, the inhibiting influence may be caused by enhanced beta-cell apoptosis and decreased transcription of the insulin gene [27]. Moreover, therapy using GCs results in hyperglycemia both in patients with diagnosed diabetes mellitus and patients without documented medical history of high glucose level occurrence before initiation of GCs usage [28]. ICI-related DM, like other ICI-related endocrinopathies, is not reversible and therefore requires lifelong insulin therapy [29].

Early diagnosis of ICI-DM allows patients to be treated more efficiently and prevent them from developing DKA or other severe side effects [23]. Therefore, the potential risk of DM and symptoms correlated to it (such as polyuria, polydipsia, thirst, dehydration, or weight loss) should be brought to patients’ and doctors’ attention. Education about the way of proceeding if these signs occur is recommended [30,31]. Moreover, control blood tests of glucose level and HbA1c are recommended to be ordered on routine visits [31].

The most frequent endocrinopathy induced by ICI is thyroid dysfunction. The mechanisms leading to this endocrinopathy are analogous to the ones described for DM; however, both CTLA-4 and PD-1 axis blockers may lead to thyroid insufficiency. Hypothyroidism management depends on the severity of the patient’s condition and their assignment to the appropriate grade [32]. If any symptoms of hypothyroidism occur, hormone replacement therapy with levothyroxine is required and might continue throughout the patient’s life. Intravenous admission of the previously mentioned drug is recommended in the case of grave symptoms, such as hypothermia or hypotension [33]. Moreover, deliberation with an endocrine team is suggested. The decision regarding discontinuation of ICI therapy should be made based on the individual patient’s condition and after multidisciplinary consultation [32]. Importantly, the appearance of a second endocrinopathy after the diagnosis of diabetes mellitus during ICI therapy was reported to be as high as 44% [34]. However, the co-development of at least two endocrinopathies was as low as 0.9%, and the sample size of the group was rather small at 27 cases included in the study [34].

## 4. Conclusions

T1DM might be a complication of immunotherapy; however, previous reports of this complication in the literature are few. Typically, it presents in a manner resembling fulminant diabetes, with a short duration of symptoms and frequent hospitalization due to ketoacidosis. A low concentration of peptide-C is typical, whereas the presence of anti-GAD antibodies occurs in only about half of the cases [35]. Early detection and better management of this complication may be achieved through vigilant monitoring for signs of diabetes. The co-appearance of two endocrinopathies during ICI treatment is not rare, so clinicians should be aware of this possibility. Especially, thorough examination for typical endocrine insufficiencies such as thyroiditis should be performed during every visit.

## Figures and Tables

**Table 1:** The incidence of the most common endocrine conditions during ICI-treatment.

Drug Name	Target	Immunoglobulin (Ig) Type	Incidence of Endocrinopathy During Treatment				
			T1DM/Hyperglycemia *	Hypothyroidism	Hyperthyroidism	Hypophysitis	Adrenalitis/Adrenal Dysfunction
Ipilimumab	CTLA-4	IgG1	No risk when administered alone	1–19% (8% overall) [9]	no data	6–30% (6% overall) [9]	1% [9]
Pembrolizumab	PD-1	IgG4	0.4% [10]	8.0% [11]	3.7% [10]	1.1% [10]	0.8% [10]
Nivolumab	PD-1	IgG4	0.9% [12]	7.6% [11]	2.8% [10]	0.5% [10]	2% [10]
Atezolizumab	PD-L1	IgG1	1.4% [10]	1.9% [11]	0.6% [13]	no data	no data
Avelumab	PD-L1	IgG1	1.1% [10]	4.6% [11]	0.6% [13]	less than 0.1% [13]	1.1% [10]
Durvalumab	PD-L1	IgG1	no data	9.0% [11]	0.6% [13]	less than 0.1% [13]	no data
Cemiplimab	PD-1	IgG4	no data	8.3% [14] *	2.8% [14]	no data	no data
Dostarlimab	PD-1	IgG4	1% [15]	11.2% [16]	3.1% [15]	0.4% [15]	1.4% [15]

* administered with chemo- or radiotherapy.

## Data Availability

No new data was created.
